# Development and clinical application of a deep learning model to identify acute infarct on magnetic resonance imaging

**DOI:** 10.1038/s41598-022-06021-0

**Published:** 2022-02-09

**Authors:** Christopher P. Bridge, Bernardo C. Bizzo, James M. Hillis, John K. Chin, Donnella S. Comeau, Romane Gauriau, Fabiola Macruz, Jayashri Pawar, Flavia T. C. Noro, Elshaimaa Sharaf, Marcelo Straus Takahashi, Bradley Wright, John F. Kalafut, Katherine P. Andriole, Stuart R. Pomerantz, Stefano Pedemonte, R. Gilberto González

**Affiliations:** 1 MGH & BWH Center for Clinical Data Science, Mass General Brigham, Boston, USA; 2 Athinoula A. Martinos Center for Biomedical Imaging, Massachusetts General Hospital, Boston, USA; 3 Harvard Medical School, Boston, USA; 4 Department of Radiology, Massachusetts General Hospital, Boston, USA; 5 Diagnósticos da América SA, São Paulo, Brazil; 6 Department of Neurology, Massachusetts General Hospital, Boston, USA; 7 GE Healthcare, Chicago, USA; 8 Department of Radiology, Brigham and Women’s Hospital, Boston, USA; 9 MGH & BWH Center for Clinical Data Science, Mass General Brigham, Suite 1303, Floor 13, 100 Cambridge St, Boston, MA 02114 USA

**Keywords:** Diagnostic markers, Magnetic resonance imaging

## Abstract

Stroke is a leading cause of death and disability. The ability to quickly identify the presence of acute infarct and quantify the volume on magnetic resonance imaging (MRI) has important treatment implications. We developed a machine learning model that used the apparent diffusion coefficient and diffusion weighted imaging series. It was trained on 6,657 MRI studies from Massachusetts General Hospital (MGH; Boston, USA). All studies were labelled positive or negative for infarct (classification annotation) with 377 having the region of interest outlined (segmentation annotation). The different annotation types facilitated training on more studies while not requiring the extensive time to manually segment every study. We initially validated the model on studies sequestered from the training set. We then tested the model on studies from three clinical scenarios: consecutive stroke team activations for 6-months at MGH, consecutive stroke team activations for 6-months at a hospital that did not provide training data (Brigham and Women’s Hospital [BWH]; Boston, USA), and an international site (Diagnósticos da América SA [DASA]; Brazil). The model results were compared to radiologist ground truth interpretations. The model performed better when trained on classification and segmentation annotations (area under the receiver operating curve [AUROC] 0.995 [95% CI 0.992–0.998] and median Dice coefficient for segmentation overlap of 0.797 [IQR 0.642–0.861]) compared to segmentation annotations alone (AUROC 0.982 [95% CI 0.972–0.990] and Dice coefficient 0.776 [IQR 0.584–0.857]). The model accurately identified infarcts for MGH stroke team activations (AUROC 0.964 [95% CI 0.943–0.982], 381 studies), BWH stroke team activations (AUROC 0.981 [95% CI 0.966–0.993], 247 studies), and at DASA (AUROC 0.998 [95% CI 0.993–1.000], 171 studies). The model accurately segmented infarcts with Pearson correlation comparing model output and ground truth volumes between 0.968 and 0.986 for the three scenarios. Acute infarct can be accurately detected and segmented on MRI in real-world clinical scenarios using a machine learning model.

## Introduction

Acute ischemic stroke is a significant cause of global morbidity and mortality^1–3^. Diffusion-weighted imaging (DWI) on magnetic resonance imaging (MRI) is highly accurate for diagnosing ischemic stroke shortly after symptom onset^4^, and the DWI lesion volume is used as a selection criterion for endovascular thrombectomy^5–9^. The selection of patients using MRI compared with CT doubles the likelihood of functional independence and results in fewer futile thrombectomies^10,11^.

Machine learning, which involves a computer mathematical model learning to perform specific tasks from existing data, is predicted to play an increasing role in clinical care^12^. It has already been applied to acute stroke computed tomography (CT) imaging interpretation including for hemorrhage identification, large vessel occlusion identification and ischemic core volume estimation^13–20^. For MRI, it has mainly been used in the context of stroke to automate segmentation of infarcted regions^21–30^. It has also been used to predict stroke onset time and hemorrhagic transformation on MRI^31–33^.

We sought to develop a machine learning model that would output the binary presence of an infarct and its segmented region. This model would therefore benefit clinicians by rapidly identifying studies with an infarct and quantifying the infarct size. In developing it, we used a combination of supervision types to leverage a greater quantity of training data^34^. We then validated this model on ‘real-world’ stroke data through identifying consecutive stroke presentations at two US academic medical centers including one that training data was not obtained from. We also validated the model on data obtained from an international site.

## Results

### Model development

**Figure 1:**
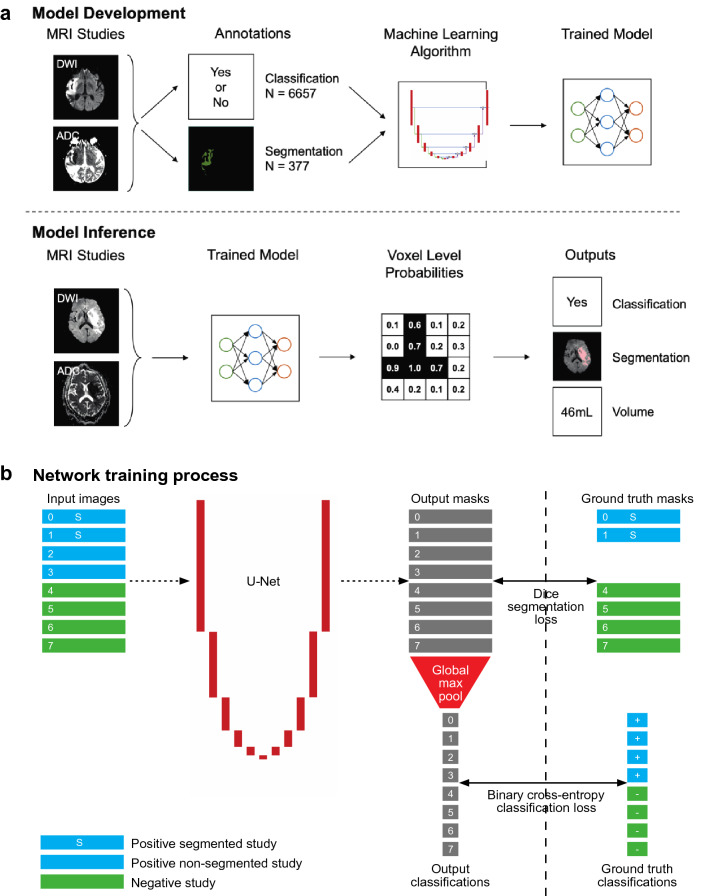
Model design and development: (**a**) The structure for development and inference of a runtime model including incorporation of both DWI and ADC sequences as well as both classification and segmentation annotations. The shading of voxel level probabilities uses an operating point of 0.5. (**b**) The training process for a single batch of DWI and ADC pairs from 8 studies. A batch consisted of 2 segmented positive studies, 2 non-segmented positive studies and 4 negative studies, which involved oversampling of segmented studies. A Dice segmentation loss was applied for the segmented positive studies and negative studies using the segmentation output masks. In addition to the segmentation output, a classification output was produced by a global max-pooling operation on the output masks. A binary cross-entropy loss was then applied for all examples in the batch using the classification output.

We used machine learning to train a model that detects acute infarct (Fig. 1; Supplementary Fig. S1). The model calculates the probability of infarct in each voxel within an MRI study. The presence of any voxel with a probability above a given operating point causes the entire study to be classified positive. The amalgamated positive voxels output as a segmented region providing infarct visualization and volume quantification.

The primary dataset consisted of MRI studies from an academic medical center and its affiliated satellites. The data were allocated to a training set, validation set and primary test set (Table 1; Supplementary Table S1 for scanner manufacturers and models). The validation set allowed optimization of model hyperparameters including selecting an appropriate operating point. We decided that an operating point of 0.5 provided an appropriate balance between sensitivity and specificity for clinical use (96.5% and 97.5% respectively on the validation set; Supplementary Table S2). We used this operating point for subsequent experiments.

**Table 1:** Dataset details: the properties of the datasets that were used for model training and testing.

	Training set		Validation set		Primary test set		Stroke code test sets		International test set
	Classification	Segmentation	Classification	Segmentation	Classification	Segmentation	Training hospital	Non-training hospital	
Number of studies	6657	377	725	34	792	62	381	247	171
Number of positive studies (%)	3314 (49.8%)	All	372 (51.3%)	All	384 (48.5%)	All	168 (44.1%)	128 (50.2%)	70 (40.9%)
Time period of studies	01/2004–05/2018	01/2004–05/2018	01/2007–05/2018	02/2007–05/2018	01/2007–05/2018	03/2007–05/2018	07/2018–01/2019	07/2018–12/2018	01/2017–07/2019
Number of studies on female patients (%)	3445 (51.8%)	176 (46.7%)	374 (51.6%)	17 (50.0%)	404 (51.0%)	26 (41.9%)	193 (50.7%)	129 (52.2%)	101 (59.1%)
Mean age in years ± standard deviation (range)	60.7 ± 18.0 (18–104)	68.1 ± 14.6 (18–102)	60.8 ± 17.7 (18–101)	67.4 ± 18.4 (26–96)	60.5 ± 18.4 (18–102)	68.2 ± 15.8 (26–99)	65.9 ± 16.5 (19–98)	67.6 ± 17.2 (22–97)	46.7 ± 21.1 (18–95)
Median infarct volume in mL (interquartile range; range)	–	6.42 (0.61–33.28; 0.02–333.06)	–	6.38 (1.42–36.03; 0.06–276.38)	–	5.57 (0.78–56.88; 0.03–308.78)	2.73 (0.47–12.98; 0.04–403.16)	6.12 (1.03–43.47; 0.10–442.80)	3.01 (0.75–14.54; 0.07–255.20)
Number of studies on GE scanners	5233	349	568	31	618	57	345	52	Unavailable
Number of studies on Siemens scanners	1424	28	157	3	174	5	36	196	Unavailable

Our machine learning architecture utilized two specific strategies to improve performance. Firstly, it included two annotation types: slice-level segmentation of the infarcted region and study-level classification of infarct presence. The segmentations provided the model with more information about individual cases but were time intensive to create, while the classification annotations exposed the model to a greater number of cases. This strategy improved AUROC for the validation set from 0.982 (95% CI 0.972–0.990) when trained on only the segmentation studies to 0.995 (95% CI 0.992–0.998) when trained on both the segmentation and classification studies (Table 2). The median Dice coefficient for overlap of ground truth and model output segmentations improved from 0.776 (interquartile range [IQR] 0.584–0.857) to 0.797 (IQR 0.642–0.861). Secondly, the model incorporated both ADC and DWI series given both are required clinically to determine restricted diffusion. The model performed with AUROC 0.954 (95% CI 0.939–0.968) on the validation set when using only ADC series, 0.991 (95% CI 0.985–0.996) when using only DWI series and 0.995 (95% CI 0.992–0.998) when using both series. The median Dice coefficient was 0.598 (IQR 0.444–0.736) with only ADC series, 0.787 (IQR 0.650–0.863) with only DWI series and 0.797 (IQR 0.642–0.861) with both series.

**Table 2:** Results summary: The model results obtained during training and testing.

	AUROC (95% CI)	Sensitivity (95% CI)	Specificity (95% CI)	Median Dice coefficient for region correlation (IQR)		Pearson coefficient for volume correlation
**Training modifications (performance on validation set)**						
Segmentation studies only (no classification studies)	0.982 (0.972–0.990)	95.4% (93.2–97.4%)	93.8% (91.1–96.2%)	0.776 (0.584–0.857)		0.988
ADC series only (no DWI series)	0.954 (0.939–0.968)	85.5% (81.8–89.0%)	95.2% (92.9–97.3%)	0.598 (0.444–0.736)		0.951
DWI series only (no ADC series)	0.991 (0.985–0.996)	95.7% (93.5–97.7%)	96.9% (94.9–98.6%)	0.787 (0.650–0.863)		0.984
Final model	0.995 (0.992–0.998)	96.5% (94.5–98.2%)	97.5% (95.6–98.9%)	0.797 (0.642–0.861)		0.987
**Test set performance**						
Primary test set	0.998 (0.995–0.999)	98.4% (97.1–99.5%)	98.0% (96.6–99.3%)	0.813 (0.727–0.863)		0.987
Training hospital stroke code test set						
GE	0.962 (0.938–0.982)	88.2% (82.7–93.1%)	95.3% (92.2–98.0%)	R1 vs M	0.726 (0.563–0.801)	0.987
				R2 vs M	0.705 (0.551–0.792)	
				R1 vs R2	0.727 (0.590–0.811)	
Siemens	0.997 (0.981–1.000)	100.0% (100.0–100.0%)	90.0% (75.0–100.0%)	R1 vs M	0.727 (0.622–0.810)	0.994
				R2 vs M	0.742 (0.594–0.802)	
				R1 vs R2	0.752 (0.634–0.838)	
Overall	0.964 (0.943–0.982)	89.3% (84.5–93.9%)	94.8% (91.7–97.6%)	R1 vs M	0.726 (0.568–0.803)	0.968
				R2 vs M	0.709 (0.551–0.793)	
				R1 vs R2	0.727 (0.598–0.813)	
Non-training hospital stroke code test set						
GE	0.988 (0.960–1.000)	100.0% (100.0–100.0%)	78.3% (60.0–94.4%)	R1 vs M	0.660 (0.509–0.811)	0.978
				R2 vs M	0.667 (0.468–0.791)	
				R1 vs R2	0.683 (0.587–0.822)	
Siemens	0.979 (0.960–0.993)	94.9% (90.2–99.0%)	88.5% (81.8–94.5%)	R1 vs M	0.649 (0.461–0.732)	0.989
				R2 vs M	0.637 (0.488–0.765)	
				R1 vs R2	0.681 (0.594–0.755)	
Overall	0.981 (0.966–0.993)	96.1% (92.3–99.2%)	86.6% (80.2–92.3%)	R1 vs M	0.658 (0.480–0.750)	0.986
				R2 vs M	0.652 (0.473–0.770)	
				R1 vs R2	0.682 (0.592–0.770)	
International test set	0.998 (0.993–1.000)	100.0% (100.0–100.0%)	98.0% (94.9–100.0%)	R1 vs M	0.686 (0.503–0.776)	0.980
				R2 vs M	0.683 (0.519–0.762)	
				R1 vs R2	0.714 (0.604–0.813)	

As there were two ground truth readers for the stroke code and international test sets, there are three Dice coefficients (reader 1 vs model [R1 vs M], reader 2 vs model [R2 vs M] and reader 1 vs reader 2 [R1 vs R2]); the Pearson coefficient for these test sets is calculated for averaged reader volume vs model volume.

**Figure 2:**
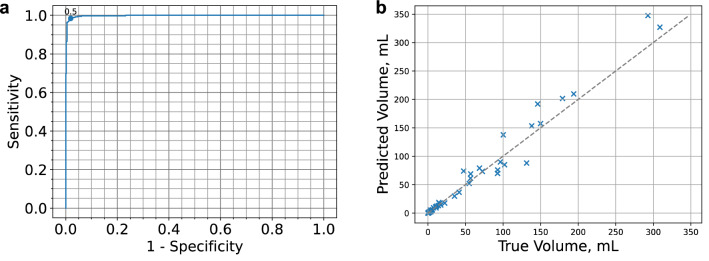
Model performance on primary test set: (**a**) Receiver operating characteristic curve for the primary test set including operating point of 0.5. (**b**) Volume plot comparing true (radiologist annotated) with predicted (model output) volumes for the primary test set.

The finalized model was evaluated on the primary test set. It performed with AUROC 0.998 (95% CI 0.995–0.999; Fig. 2a), sensitivity 98.4% (95% CI 97.1–99.5%) and specificity 98.0% (95% CI 96.6–99.3%) for infarct detection. The median Dice coefficient was 0.813 (IQR 0.727–0.863) and the Pearson correlation coefficient for the segmentation volumes was 0.987 (Fig. 2b). The inference time was less than 20 s for all studies in the primary test set and subsequent test sets.

### Stroke code test set performance

As balanced datasets can differ from real-world clinical scenarios, the model was next evaluated on MRI studies performed after ‘stroke code’ activations^12^. These activations reflect group pager messages to mobilize team members including neurology, radiology and pharmacy after a patient presents with stroke symptoms. Approximately half of these patients ultimately have an infarct. We obtained the activations over a six-month period from two hospitals including the hospital that training data was obtained from (‘training hospital’) and a hospital that training data was not obtained from (‘non-training hospital’).

**Figure 3:**
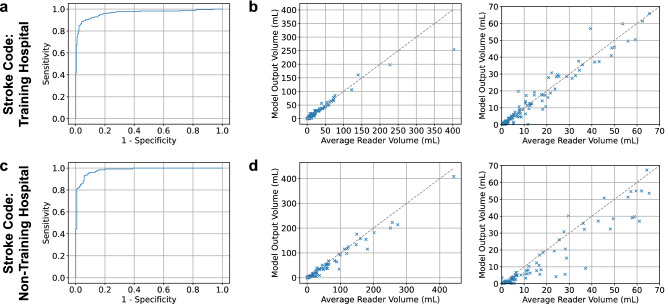
Model performance on stroke code test sets: (**a**, **b**) Training hospital stroke code receiver operating characteristic curve (**a**) and volume plot comparing averaged reader volume with model output volume (**b**; with magnified view of 0–70 mL on the right). (**c**, **d**) Non-training hospital stroke code receiver operating characteristic curve (**c**) and volume plot comparing averaged reader volume with model output volume (**d**; with magnified view of 0–70 mL on the right).

The training hospital had 598 stroke codes for which 396 MRI studies occurred and 381 met model inclusion criteria (Supplementary Fig. S2; Supplementary Table S3 for manufacturers and models). There were 168 positive studies (44.1%). The model performed with AUROC 0.964 (95% CI 0.943–0.982), sensitivity 89.3% (95% CI 84.5–93.9%) and specificity 94.8% (95% CI 91.7–97.6%) for classification (Fig. 3a). The model also outputted segmented infarct regions (Supplementary Fig. S3). The model volume quantification had Pearson correlation 0.968 compared with the averaged reader volume (Fig. 3b and Supplementary Fig. S4). The Bland–Altman analysis between the averaged reader and model volumes provided a difference of − 0.4 mL (95% CI − 6.9 to + 6.1 mL) for infarcts less than 70 mL and − 1.5 mL (95% CI − 27.0 to + 24.0 mL) for all infarcts (Supplementary Fig. S5). The overlap of segmented regions was similar for the model compared to each reader as it was between readers: the median Dice coefficient was 0.726 (IQR 0.568–0.803) for model versus reader 1, 0.709 (IQR 0.551–0.793) for model versus reader 2, 0.727 (IQR 0.598–0.813) for reader 1 versus reader 2. The model performed similarly on GE and Siemens scanners with AUROCs 0.962 (95% CI 0.938 to 0.982) and 0.997 (95% CI 0.981 to 1.000) respectively. While two patients were excluded from the final analysis due to age < 18 years, the model correctly predicted both studies as negative.

The non-training hospital had 494 stroke codes for which 255 MRI studies occurred and 247 met model inclusion criteria (Supplementary Fig. S6; Supplementary Table S3 for manufacturers and models). There were 128 positive studies (51.8%). The model performed with AUROC 0.981 (95% CI 0.966–0.993), sensitivity 96.1% (95% CI 92.3–99.2%) and specificity 86.6% (95% CI 80.2–92.3%) for classification (Fig. 3c). The model volume quantification had Pearson correlation 0.986 compared with the averaged reader volume (Fig. 3d; Supplementary Fig. S7). The Bland–Altman analysis between the averaged reader and model volumes provided a difference of − 3.1 mL (95% CI, – 14.4 to + 8.2 mL) for infarcts less than 70 mL and − 6.1 mL (95% CI − 31.2 to + 19.0 mL) for all infarcts (Supplementary Fig. S8). The overlap of segmented regions was similar for the model compared to each reader as it was between readers: the median Dice coefficient was 0.658 (IQR 0.480–0.750) for model versus reader 1, 0.652 (IQR 0.473–0.770) for model versus reader 2, 0.682 (IQR 0.592–0.770) for reader 1 versus reader 2. The model performed similarly on GE and Siemens scanners with AUROCs 0.988 (95% CI 0.960 to 1.000) and 0.979 (95% CI 0.960 to 0.993) respectively.

**Figure 4:**
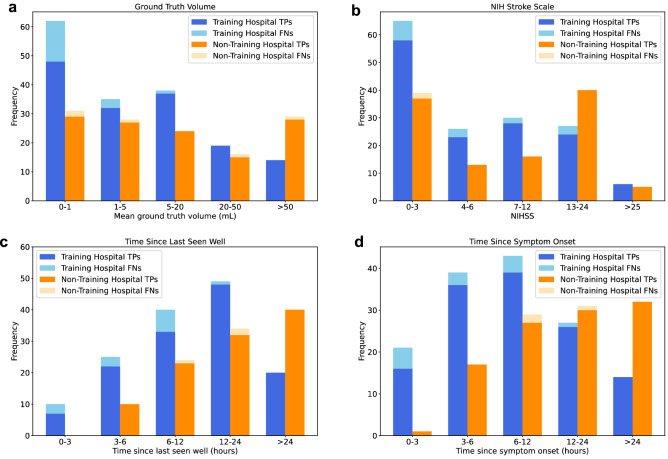
Model performance based on clinical scenario for stroke code test sets: (**a**–**d**) Histograms demonstrating the number of true positive (TP) and false negative (FN) studies for different ground truth volumes (**a**), NIH Stroke Scales (**b**), time intervals between last seen well and MRI (**c**), and time intervals between symptom onset and MRI (**d**). The images from the false negative studies with ground truth volume > 1 mL are included in Supplementary Fig. S9. As an example of the time intervals, a patient who presents at 8 am having gone to sleep without symptoms at 10 pm and woken with symptoms at 6 am will have time from last seen well of 10 h and time from symptom onset of 2 h.

We reviewed the false negative and false positive studies from the training hospital and non-training hospital. The majority of false negative studies were for infarcts that were less than 1 mL (14 out of 18 studies at the training hospital and 2 out of 5 studies at the non-training hospital; Fig. 4a and Supplementary Fig. S9a). The remaining false negative studies were felt secondary to subtle ADC hypointensity (4 studies) and atypical infarcts (1 study for each of air embolism etiology, venous etiology and atypical hippocampal location; all studies displayed in Supplementary Fig. S9b–e). Overall false negative studies had smaller infarct sizes compared to true positive studies (mean averaged reader volume 12.3 mL for false negatives and 26.4 mL for true positives; Spearman correlation between classification probability and volume of 0.764, p < 0.001; Fig. 4a). The false positive studies mostly reflected ‘mimics' of acute infarct including hemorrhage and tumor (Supplementary Fig. S10). We found one ‘false positive' punctate infarct that the readers labelled negative, but on review was more evident on an MRI performed three days later and should have been labelled positive (Supplementary Fig. S10d); its ground truth was not updated given the ground truth interpretations were locked prior to comparison with model outputs.

We also obtained the National Institutes of Health Stroke Scale (NIHSS), last seen well time (when a patient last had no symptoms) and symptom onset time (when a patient first had symptoms) for patients with an infarct, in order to stratify model performance by these clinical variables (Fig. 4b–d). Overall false negative studies were more likely to have a lower NIHSS (average NIHSS 5.1 for false negative studies and 8.7 for true positive studies; Spearman correlation between classification probability and NIHSS of 0.442, p < 0.001), shorter duration between the MRI and last seen well time (average interval 8.2 h for false negative studies and 17.5 h for true positive studies; Spearman correlation 0.291, p < 0.001), and shorter duration between the MRI and symptom onset time (average interval 6.8 h for false negative studies and 14.4 h for true positive studies; Spearman correlation 0.271, p < 0.001).

### International test set performance

**Figure 5:**
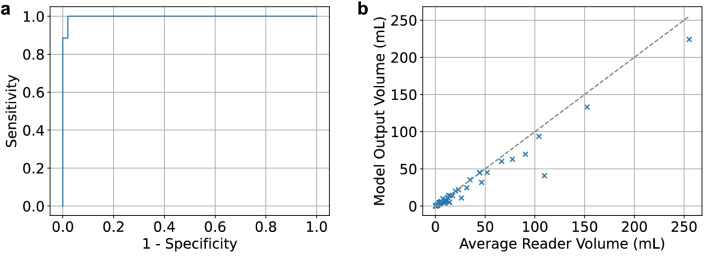
Model performance on international test set: (**a**) Receiver operating characteristic curve for the international test set. (**b**) Volume plot comparing averaged reader volume with model output volume for the international test set.

To further demonstrate the generalizability of our model, we tested it on 171 MRI studies, including 70 positive studies (40.9%), obtained from Brazil. The initial dataset contained an additional 6 studies that were excluded (2 with no DWI/ADC series; 4 non-diagnostic with significant motion or metal artifact). The model performed with AUROC 0.998 (95% CI 0.993–1.000), sensitivity 100% (95% CI 100–100%) and specificity 98.0% (95% CI 94.9–100%) for classification (Fig. 5a). The model volume quantification had Pearson correlation 0.980 compared with the averaged reader volume (Fig. 5b; Supplementary Fig. S11). The Bland–Altman analysis between the averaged reader and model volumes provided a difference of − 1.6 mL (95% CI − 8.1 to + 4.9 mL) for infarcts less than 70 mL and − 3.9 mL (95% CI − 23.1 to + 15.4 mL) for all infarcts (Supplementary Fig. S12). The overlap of segmented regions was similar for the model compared to each reader as it was between readers: the median Dice coefficient was 0.686 (IQR 0.503–0.776) for model versus reader 1, 0.683 (IQR 0.519–0.762) for model versus reader 2, 0.714 (IQR 0.604–0.813) for reader 1 versus reader 2.

## Discussion

We sought to develop a machine learning algorithm that would both detect and segment acute infarcts on MRI imaging. We then demonstrated the effectiveness of this algorithm in three clinical scenarios including two stroke code test sets (at training and non-training hospitals) and an international test set.

The majority of published MRI acute infarct machine learning models focus on segmentation with Dice coefficients between 0.57 and 0.86^21–30,35^. Our model performed similarly with median Dice coefficients between 0.652 and 0.813 for the four test sets﻿. Importantly, the median Dice coefficients between our model and radiologists were also within 5% of the median inter-reader Dice coefficients suggesting concordance with radiologist segmentations. Classification is reported less frequently amongst published models; a recent study reported sensitivity 91% and specificity 75% while our model had sensitivity between 89.3 and 100.0%, and specificity between 86.6% and 98.0% for the test sets^35^.

A key clinical benefit of this model is rapid interpretation of MRI studies performed for acute stroke to enable rapid treatment decisions. This interpretation includes infarct volume quantification, which is a selection criterion for extended window endovascular thrombectomy^7,8^. The model may also be used for real-time study interpretation to prioritize studies for interpretation by a radiologist or to suggest additional studies, such as magnetic resonance angiography, while a patient remains in an MRI scanner. A possible future application involves registering the infarcted regions to an anatomic atlas to investigate whether infarcts in specific brain regions impact prognosis.

This paper used two types of annotation as part of the machine learning model design. The slice-level infarct segmentations were time intensive to create but provided the model with explicit regions of interest from which to learn. The study-level classifications took less time to create and could be performed for a greater number of studies. The classification performance of the model improved when classification annotations were included in addition to segmentations (AUROC 0.982 to 0.995 on the validation set). Remarkably the segmentation performance of the model also improved (median Dice coefficient 0.776 to 0.797) without the addition of further segmentation studies. This strategy could be further explored for the development of imaging-based algorithms to overcome extensive annotation needs, which are often a rate-limiting step.

Another important strategy involved using both the DWI and ADC series, as others have also reported^24,30^. In our results, it improved both the classification and segmentation performance when compared to only one of these series. We see this technique of combining spatially aligned series that provide complementary information as being crucial for providing more complex machine learning interpretations of MRI studies. For example, evaluation of tumors could benefit from interpretation of T2-FLAIR and T1 post-contrast series concurrently for better differentiation of findings such as edema and necrosis.

One of the biggest limitations with machine learning models is their ability to generalize to new types of data including geography, demography and technical parameters (such as scanner manufacturer and model). We sought to address this concern through demonstrating the model performance on an international test set. The model actually performed better on this test set, which we attribute to fewer small strokes (with subsequent fewer false negatives) and fewer stroke mimics (with subsequent fewer false positives) compared to the stroke code test sets. Prior to clinical use, the model will require more rigorous evaluation on further data in addition to obtaining market clearance by global regulators.

## Conclusions

This model provides accurate detection and segmentation of acute infarct that should enhance the interpretation of MRI studies in the acute stroke clinical environment.

## Methods

### Institutional review board

This retrospective study was HIPAA-compliant and approved with waived patient consent by the Partners HealthCare System (now Mass General Brigham) Institutional Review Board and the Centralized Brazilian Human Research Ethics Evaluation System (Plataforma Brasil) through Hospital Geral do Grajaú—Associação Congregação de Santa Catarina. This study was conducted in accordance with relevant local guidelines and regulations.

### Primary dataset

The cohort for model development was identified by searching for MRI brain studies in the radiology archive at a US academic medical center, which was a regional telestroke network hub, and its affiliated satellite locations. The study reports were parsed using natural language processing to identify studies that were positive or negative for acute infarct. Parsing methods included keyword and sentence matching, and creation of a simple text classifier based on N-grams and Support Vector Machines. The axial DWI B-1000 and ADC series with slice thickness ≤ 5 mm were isolated using an early version of a published series selection tool (criteria in Supplementary Table S4)^36^. A radiologist then assessed all studies to ensure correct binary classification (for infarct presence or absence) and appropriate series selection. Studies were excluded if they were non-diagnostic (for example, due to severe metal or motion artifact) or contained acute infarct mimics (non-ischemic causes of restricted diffusion including hemorrhage). The primary dataset was split approximately 80/10/10% into training, validation and test sets. This split was performed randomly, although a small number of > 70 mL infarcts were later added to only the test set to better assess its accuracy in detecting infarcts > 70 mL (the test set had a total of 14 infarcts > 70 mL). The dataset demographics are summarized in Table 1.

A subset of studies with infarct from the primary dataset underwent manual segmentation of the restricted diffusion region. Radiologists performed these segmentations on individual axial slices using Osirix MD version 9.0 or above (https://www.osirix-viewer.com/). Both the DWI and ADC series were used for the identification of restricted diffusion, with prioritization of the ADC series given possible T2 shine-through effects on DWI. Segmentations were converted into Neuroimaging Informatics Technology Initiative (NIfTI) masks for machine learning algorithm training and testing.

### Stroke code test sets

Two test sets were created from stroke team activations (‘stroke codes’) at two US academic medical centers that were hubs for regional telestroke networks. One of these academic medical centers was the source of the primary dataset (‘training hospital’) and the other was not (‘non training hospital’). All consecutive stroke codes between July 1 2018 and December 31 2018 were identified using pager system records. Individual pager messages were matched to medical record numbers (MRNs) using radiologic and clinical records. The MRNs were then matched to MRI brain studies that occurred from 1 h before to 3 days after the pager message was sent. For patients with multiple studies, the first study during the time period was used. Clinical data were acquired from the electronic medical record. Studies underwent the same series selection process as the primary dataset. Each study was separately annotated by two radiologists. The annotations included classification for presence or absence of acute infarct, and segmentation of the infarct region when present. When the two radiologists disagreed on the classification of a study, the study was reviewed by a third radiologist who made the final decision; if a classification changed from the absence to presence of acute infarct then the original radiologist who had marked the study as negative was asked to reassess the image and segment the infarct region. The annotations used the same software and file formats as the primary dataset.

### International test set

The international test set was obtained from two large hospitals in Brazil. The studies, which were also used for a separate CT model (results unpublished), were identified by searching for paired head CTs and MRIs between 2017 and 2019. Studies were selected using a natural language processing tool if the report included clinical suspicion for stroke or finding of acute infarct. The report and images were reviewed by a radiologist for appropriateness. Studies were annotated by two neuroradiologists with a third arbitrating in a similar manner to the stroke code test sets.

### Model development

The neural network architecture used was based on the popular 3D UNet segmentation model (Supplementary Fig. S1)^37,38^. The input to the network was an array consisting of two channels that contained the DWI and ADC series. Each series was resized to 256 × 256 × *n* as appropriate where *n* was the original number of slices in the acquired series. The sizes of the convolutional layers are provided in Supplementary Fig. S1. The convolutional layers, with the exception of the output convolutional layer, used a 3 × 3 × 3 kernel and were followed by both a batch normalization layer and “leaky ReLU” activation function with α slope coefficient of 0.3^39^. The output convolutional layer used a 1 × 1 × 1 kernel and was followed by a sigmoid activation function. The output of the network was a 256 × 256 × *n* segmentation mask. During downsampling, the image was max-pooled by a factor of 2 in the *x* and *y* dimensions (the two dimensions within the axial imaging plane); the full resolution in the *z* dimension was maintained. The reverse pattern was used for the upsampling layers. The network could therefore function on images with an arbitrary number of axial slices, avoiding resampling in the *z* dimension which otherwise was found to make small infarcts less obvious. The classification prediction for the study, which constituted a second network output, was found with a global max-pooling operation on the segmentation mask (i.e. if any pixel in the segmentation mask was positive then the entire study was considered positive for acute infarct). The volume prediction for the study was calculated from the aggregation of positive pixels in the segmentation mask using the pixel dimensions.

The loss function used for training the model consisted of two terms, which were summed together with equal weight. The first term was a binary cross-entropy loss function applied between the network classification output and the ground truth classification label (negative or positive for infarct). The second term was a soft Dice coefficient loss function applied between the network output segmentation mask and the ground truth segmentation mask^40^. The second term was only applied to negative training studies (which were known to have empty segmentation masks) and the segmented subset of the positive training studies.

The batch size during the training process was 8 pairs of DWI/ADC series. During training, the batches were balanced such that every batch contained 2 positive studies with segmentation masks, 2 positive studies without segmentation masks and 4 negative studies. This balancing was found to stabilize training. The Adam optimizer was used with an initial learning rate of 1 × 10^–4^^41^. Training was run for 200 epochs with the learning rate reduced by a factor of 10 after 100 epochs and by another factor of 10 after a further 50 epochs.

In order to normalize the pixel intensities for each series, the 3rd and 97th percentiles of the intensity distribution were calculated (denoted *I*_3_ and *I*_97_ respectively). The intensities were then mapped according to the following equations:

$${I}_{min}={I}_{3}$$

$${I}_{max}= {I}_{97}+ \alpha ({I}_{97}-{I}_{3})$$

$${I}_{out}=\left\{\begin{array}{ll}{I}_{min},& {\mathrm{if}} {I}_{in}<{I}_{min}\\ {I}_{max},& {\mathrm{if}} {I}_{in}>{I}_{max}\\ \frac{{I}_{in}-{I}_{min}}{{I}_{max}-{I}_{min}},& {\mathrm{otherwise}}\end{array}\right.$$

The equations used α as a constant value that was set, following initial experimentation, to α = 0 for ADC series and α = 1 for DWI series in order to ensure that areas of restricted diffusion with high pixel intensities were not saturated.

During training, augmentation was applied to the images in the form of random small rotations up to 10° in either direction within the *x–y* plane, random small translations of up to 10% of the image dimensions in the *x* and *y* directions, and scaling by a random factor between 0.9 and 1.1 in the *x* and *y* directions. Additionally, a random offset of up to 0.2(*I*_97_* – I*_3_) in either direction was applied to the *I*_*min*_ and *I*_*max*_ values used to scale the intensities.

Rather than train a 3D segmentation network from random initialization, a pre-training step on a 2D segmentation network was employed. The 2D network used the same network architecture except for having 2D convolution kernels instead of 3D kernels. Batches consisted of all axial slices from 16 pairs of DWI/ADC series and the optimizer used an initial learning rate of 1 × 10^–3^. The dataset for this pre-training consisted of all positive segmented studies and all negative studies but omitted positive non-segmented studies because labels at the level of a single slice were not available for them. A single training epoch consisted of all positive segmented studies and an equal number of negative studies randomly selected from the full set of negative studies at the start of the epoch. The training procedure was otherwise identical to the 3D network. After the pre-training step, the 2D convolution kernels provided the initialization values for the weights of the central axial plane in the 3D convolution kernels; the initialization values for the weights of the other planes were set to 0. The batch normalization parameters and convolution bias terms were also initialized from the 2D network.

The network and training process were implemented using the Keras deep learning framework with the Tensorflow backend. Training was performed with 4 Nvidia V100 Graphics Processing Units (GPUs). The architecture was finalized after evaluation of different architectures and training parameters on the validation set.

### Evaluation of the algorithm

Evaluation of the model classification output was performed by examining the AUROC, and sensitivity and specificity using specified operating points. The 95% confidence interval (95% CI) was calculated using a bootstrapping method with 10,000 iterations. Evaluation of the model segmentation output was performed on true positive studies (i.e. positive for radiologist and model outputs) by examining the Dice coefficient for overlap of segmentation output, and the Pearson correlation coefficient and Bland Altman analysis for volume output. Comparison of infarct volume and clinical variables between true positive and false negative studies was performed by plotting the relevant variable against the classification probability for the study (≥ 0.5 for true positives and < 0.5 for false negatives), then calculating the Spearman rank correlation coefficient; data from the training and non-training hospitals were grouped together for this analysis.

## Supplementary Information

Supplementary Information.

## Data Availability

The data used for the primary dataset, stroke code test sets and international test were obtained from hospitals as described above. Data use was approved by relevant institutional review boards. The data are not publicly available and restrictions apply to their use.

## Code availability

The model and related source code are proprietary and will not be publicly released. All experiments are described in sufficient detail in the “Methods” to enable independent replication.
