# Supplementary material for: Development and clinical application of a deep learning model to identify acute infarct on magnetic resonance imaging
Source: Sci Rep. 2022 Feb 9;12:2154. doi: 10.1038/s41598-022-06021-0 (PMC8828773; doi:10.1038/s41598-022-06021-0)
Supplement: Supplementary file 1 — Supplementary Information. [file 41598_2022_6021_MOESM1_ESM.docx]

**Online-only material**

[Supplementary Table S1 2](#_Toc88082358)

[Supplementary Table S2 3](#_Toc88082359)

[Supplementary Table S3 4](#_Toc88082360)

[Supplementary Table S4 5](#_Toc88082361)

[Supplementary Figure S1 6](#_Toc88082362)

[Supplementary Figure S2 7](#_Toc88082363)

[Supplementary Figure S3 8](#_Toc88082364)

[Supplementary Figure S4 9](#_Toc88082365)

[Supplementary Figure S5 10](#_Toc88082366)

[Supplementary Figure S6 11](#_Toc88082367)

[Supplementary Figure S7 12](#_Toc88082368)

[Supplementary Figure S8 13](#_Toc88082369)

[Supplementary Figure S9 14](#_Toc88082370)

[Supplementary Figure S10 15](#_Toc88082371)

[Supplementary Figure S11 16](#_Toc88082372)

[Supplementary Figure S12 17](#_Toc88082373)

# Supplementary Table S1

**Primary dataset scanner manufacturers and models:** Details of scanner manufacturers and models amongst the primary dataset.

|  |  |  |  |  | Training set | | Validation set | | Primary test set | |
| --- | --- | --- | --- | --- | --- | --- | --- | --- | --- | --- |
| Manufacturer | Model | Field strength (T) | Gradient slew rate (T/m/s) | Gradient strength (mT/m) | Classification | Segmentation | Classification | Segmentation | Classification | Segmentation |
| GE | DISCOVERY MR750 | 3 | 200 | 50 | 307 | 2 | 32 | 0 | 32 | 0 |
| GE | GENESIS_SIGNA | 1.5 | Unavailable | | 1 | 1 | 0 | 0 | 0 | 0 |
| GE | Optima MR450w | 1.5 | 200 | 44 | 644 | 62 | 58 | 4 | 85 | 12 |
| GE | SIGNA Artist | 1.5 | 80 & 150 | 40 | 270 | 22 | 33 | 4 | 4 | 4 |
| GE | SIGNA EXCITE | 1.5 | Unavailable | | 3 | 0 | 0 | 0 | 1 | 0 |
| GE | SIGNA HDx | 1.5 | 120 | 40 | 688 | 147 | 90 | 14 | 83 | 23 |
| GE | SIGNA HDxt | 1.5 | 80 & 150 | 40 | 3320 | 115 | 355 | 9 | 413 | 18 |
| GE | Total |  |  |  | 5233 | 349 | 568 | 31 | 618 | 57 |
| Siemens | Aera | 1.5 | 200 | 45 | 5 | 0 | 0 | 0 | 0 | 0 |
| Siemens | Avanto | 1.5 | 200 | 45 | 367 | 0 | 34 | 0 | 45 | 0 |
| Siemens | Avanto fit | 1.5 | 200 | 45 | 63 | 0 | 5 | 0 | 6 | 0 |
| Siemens | Espree | 1.5 | Unavailable | | 1 | 0 | 0 | 0 | 1 | 0 |
| Siemens | Prisma fit | 3 | 200 | 45 | 161 | 2 | 28 | 1 | 24 | 1 |
| Siemens | Skyra | 3 | 200 | 45 | 505 | 22 | 54 | 2 | 72 | 4 |
| Siemens | SymphonyTim | 1.5 | Unavailable | | 1 | 0 | 0 | 0 | 0 | 0 |
| Siemens | TrioTim | 3 | 200 | 45 | 318 | 4 | 36 | 0 | 25 | 0 |
| Siemens | Verio | 3 | 200 | 45 | 3 | 0 | 0 | 0 | 1 | 0 |
| Siemens | Total |  |  |  | 1424 | 28 | 157 | 3 | 174 | 5 |

# Supplementary Table S2

**Operating point determination:** Example operating points from validation set receiver operating characteristic curve. The chosen operating point was 0.5.

| Operating point | Sensitivity | Specificity |
| --- | --- | --- |
| 0.01 | 98.9% | 86.1% |
| 0.05 | 97.8% | 94.1% |
| 0.2 | 97.3% | 96.6% |
| 0.5 | 96.5% | 97.5% |
| 0.8 | 95.7% | 98.3% |
| 0.95 | 94.9% | 100% |
| 0.99 | 91.9% | 100% |

# Supplementary Table S3

**Stroke code manufacturers and models:** Details of scanner manufacturers and models amongst the stroke code datasets.

| Manufacturer | Model | Field strength (T) | Gradient slew rate (T/m/s) | Gradient strength (mT/m) | Training Hospital | Non-Training Hospital |
| --- | --- | --- | --- | --- | --- | --- |
| GE | DISCOVERY MR750w | 3 | Unavailable | |  | 52 |
| GE | SIGNA Artist | 1.5 | 80 & 150 | 40 | 333 |  |
| GE | Signa HDxt | 1.5 | 80 & 150 | 40 | 12 |  |
| GE | Total |  |  |  | 345 | 52 |
| Siemens | Aera | 1.5 | 200 | 45 |  | 147 |
| Siemens | Prisma_fit | 3 | 200 | 45 | 1 |  |
| Siemens | Skyra | 3 | 200 | 45 | 35 |  |
| Siemens | Verio | 3 | 200 | 45 |  | 49 |
| Siemens | Total |  |  |  | 36 | 196 |

# Supplementary Table S4

**Criteria for series selection:** Studies included in both training and test sets needed to contain a DWI sequence and ADC sequence that met these criteria.

| Characteristic | Allowed Values |
| --- | --- |
| Diffusion B value | 1000 |
| Slice thickness | 4.0mm to 5.0mm  (4.0mm to 6.0mm for international dataset) |
| Pulse sequence | 'ep_b1000t', '*ep_b1000t', 'r1_b1000t', '*re_b1000t' (Siemens DWI Images)  'ep_b0_1000', '*ep_b0_1000', 'r1_b0_1000', '*re_b0_1000' (Siemens ADC Images)  'epi2' (GE Images) |
| Series acquisition time | DWI and ADC sequences have recorded acquisition times within 10 seconds of each other. |

# Supplementary Figure S1

**Model architecture:** Diagram demonstrating model architecture. The input for the model consisted of DWI and ADC series that included 256 × 256 pixel images (resized if necessary) with *n* slices (approximately 20-30). These series were concatenated to form two input channels for the model with total shape 256 × 256 × *n* × 2. The model architecture consisted of a series of convolutional layers (red boxes) with the output shapes of each shown underneath the box. The output of the model was a segmentation mask of size 256 × 256 × *n* × 1 in which each pixel was classified as being positive or negative for acute infarct.

# Supplementary Figure S2

**Training hospital stroke code test set:** The selection of studies from stroke code activations at the training hospital.

# Supplementary Figure S3

**Training hospital segmentations:** Example images of true positive studies. Each study includes DWI, ADC, DWI with model output segmentation (in red) and DWI with ground truth segmentation (in green) sequences.

# Supplementary Figure S4

**Training hospital volume curves:** Graphs that compare the volume outputs from segmentations of Model and Reader 1 (**a**), Model and Reader 2 (**b**), and Reader 1 and Reader 2 (**c**). The graphs on the right provide magnified view of 0-70mL.

# Supplementary Figure S5

**Training hospital Bland-Altman analysis:** Graphs that compare the volume outputs of Model and Average of Reader 1 and 2 (**a**), Model and Reader 1 (**b**), Model and Reader 2 (**c**), and Reader 1 and Reader 2 (**d**). The graphs on the right provide magnified view of 0-70mL.

# Supplementary Figure S6

**Non-training hospital stroke code test set:** The selection of studies from stroke code activations at the non-training hospital.

# Supplementary Figure S7

**Non-training hospital volume curves:** Graphs that compare the volume outputs from segmentations of Model and Reader 1 (**a**), Model and Reader 2 (**b**), and Reader 1 and Reader 2 (**c**). The graphs on the right provide magnified view of 0-70mL.

# Supplementary Figure S8

**Non-training hospital Bland-Altman analysis:** Graphs that compare the volume outputs of Model and Average of Reader 1 and 2 (**a**), Model and Reader 1 (**b**), Model and Reader 2 (**c**), and Reader 1 and Reader 2 (**d**). The graphs on the right provide magnified view of 0-70mL.

# Supplementary Figure S9

**False negative studies in the stroke code test sets: a**, Suspected reasons for false negative studies in the stroke code tests sets. **b-e**, Images of false negative studies demonstrating subtle ADC hypointensity (**b**), air embolism infarct (**c**), venous infarct (**d**) and atypical hippocampal infarct (**e**). Each study includes DWI, DWI with ground truth segmentation (in green) and ADC sequences.

# Supplementary Figure S10

**False positive studies in the stroke code test sets: a**, Suspected reasons for false positive studies in the stroke code tests sets. * denotes one study had two regions that were annotated as positive by the model with different reasons. **b-f**, Images of false positive studies demonstrating hemorrhage (**b**), tumor (**c**), early infarct (**d**), chronic infarct (**e**), motion (**f**) and punctate DWI hyperintensity (**g**). Each study includes DWI, DWI with model segmentation (in red) and ADC sequences.

# Supplementary Figure S11

**International test set volume curves:** Graphs that compare the volume outputs from segmentations of Model and Reader 1 (**a**), Model and Reader 2 (**b**), and Reader 1 and Reader 2 (**c**). The graphs on the right provide magnified view of 0-70mL.

# Supplementary Figure S12

**International test set Bland-Altman analysis:** Graphs that compare the volume outputs of Model and Average of Reader 1 and 2 (**a**), Model and Reader 1 (**b**), Model and Reader 2 (**c**), and Reader 1 and Reader 2 (**d**). The graphs on the right provide magnified view of 0-70mL.
